# 42. INSPIRE-ASP UTI Trial: A 59 Hospital Cluster Randomized Evaluation of INtelligent Stewardship Prompts to Improve Real-time Empiric Antibiotic Selection versus Routine Antibiotic Selection Practices for Patients with Urinary Tract Infection (UTI)

**DOI:** 10.1093/ofid/ofab466.244

**Published:** 2021-12-04

**Authors:** Shruti K Gohil, Edward Septimus, Ken Kleinman, Neha Varma, Lauren Heim, Syma Rashid, Risa Rahm, William S Cooper, Laura E McLean, Naoise G Nickolay, Robert A Weinstein, Edward Rosen, Taliser R Avery, Sljivo Selsebil, Justin Vigeant, Kenneth Sands, Mandelin Cooper, H L Burgess, Julia Moody, Micaela H Coady, Gilbert F Rebecca, Kimberly N Smith, Brandon Carver, Caren Spencer-Smith, Russell Poland, Jason Hickok, S G Sturdevant, Anastasiia Weiland, Abinav Gowda, Robert Wolf, Mary K Hayden, Sujan Reddy, Melinda M Neuhauser, Arjun Srinivasan, Arjun Srinivasan, David W Kubiak, John A Jernigan, John A Jernigan, Jonathan B Perlin, Richard Platt, Susan S Huang

**Affiliations:** 1 UC Irvine School of Medicine, IRVINE, California; 2 Harvard Medical School, Houston, Texas; 3 University of Massachusetts, Amherst, Massachusetts; 4 HCA Healthcare, Nashville, Tennessee; 5 Rush University Medical Center, Chicago, IL; 6 Harvard Pilgrim Healthcare Institute, Boston, Massachusetts; 7 Ondine, Nashville, Tennessee; 8 NIH, Baltimore, Maryland; 9 UC Irvine, Irvine, California; 10 Boston University School of Medicine, Boston, California; 11 Centers for Disease Control and Prevention, Atlanta, GA; 12 Brigham and Women’s Hospital, Boston, Massachusetts; 13 University of California, Irvine, Irvine, CA

## Abstract

**Background:**

Up to 40% of hospitalized patients receive unnecessary or inappropriately broad antibiotics despite a low risk of multidrug-resistant organism (MDRO) infection. Empiric standard spectrum antibiotic use would reduce extended-spectrum (ES) antibiotic exposure and future resistance. We evaluated whether computerized prescriber order entry prompts providing patient-specific MDRO risk estimates could reduce ES antibiotic use compared to routine stewardship practices in patients hospitalized with urinary tract infection (UTI).

**Methods:**

This 59-hospital cluster randomized trial compared: 1) INSPIRE prompts providing patient-specific MDRO UTI risk estimates at order entry and recommended standard spectrum antibiotics for risk < 10% versus 2) routine stewardship practices. Prompt used an absolute MDRO risk algorithm based on a 140 hospital data set. Trial population included adults treated with antibiotics for UTI in ED or non-ICU wards in first 3 days of admission (empiric days); prompt was triggered if ES antibiotics were ordered. Prescribers received feedback on prompt response. Trial periods: 18-month Baseline (Apr 2017–Sept 2018); 6-month Phase-in (Oct 2018–Mar 2019); 15-month Intervention (Apr 2019 – June 2020). Primary outcome was ES antibiotic days of therapy (ES-DOT) per empiric day; secondary outcomes were a) vancomycin and b) anti-pseudomonal DOT per empiric day. Unadjusted, as-randomized analyses used generalized linear mixed effects models to assess differences in ES-DOT rates between the intervention vs baseline period across arms (difference in differences), while clustering by patient and hospital.

**Results:**

**Results:** We randomized 59 hospitals in 12 states, with 87,749 and 66,996 non-ICU UTI admissions in baseline and intervention periods, respectively. Intervention group had a a 21% reduction in ES-DOT compared to routine care. Vancomycin and anti-pseudomonal DOT were similarly reduced in the intervention group by 17% and 23%, respectively (Table).

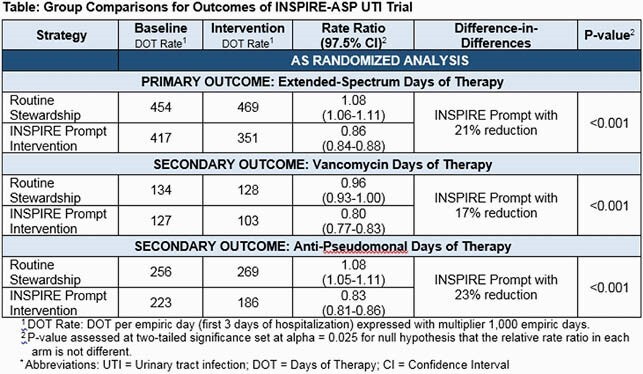

**Conclusion:**

**Conclusion:** INSPIRE order entry prompts providing real-time, patient-specific MDRO risk estimates with recommendation to use standard spectrum antibiotics in low risk patients significantly reduced empiric ES prescribing in adults admitted with UTI.

**Disclosures:**

**Shruti K. Gohil, MD, MPH**, **Medline** (Other Financial or Material Support, Co-Investigator in studies in which participating hospitals and nursing homes received contributed antiseptic and cleaning products)**Molnycke** (Other Financial or Material Support, Co-Investigator in studies in which participating hospitals and nursing homes received contributed antiseptic and cleaning products)**Stryker (Sage**) (Other Financial or Material Support, Co-Investigator in studies in which participating hospitals and nursing homes received contributed antiseptic and cleaning products) **Edward Septimus, MD**, **Medline** (Other Financial or Material Support, Conducted studies in which participating hospitals received contributed antiseptic products)**Molnlycke** (Other Financial or Material Support, Conducted studies in which participating hospitals received contributed antiseptic products) **Ken Kleinman, PhD**, **Medline** (Other Financial or Material Support, Conducted studies in which participating hospitals received contributed antiseptic products)**Molnlycke** (Other Financial or Material Support, Conducted studies in which participating hospitals received contributed antiseptic products) **Lauren Heim, MPH**, **Medline** (Other Financial or Material Support, Conducted clinical trials and studies in which participating hospitals and nursing homes received contributed antiseptic and cleaning products)**Molnlycke** (Other Financial or Material Support, Conducted studies in which participating hospitals received contributed antiseptic product)**Stryker (Sage**) (Other Financial or Material Support, Conducted clinical trials and studies in which participating hospitals and nursing homes received contributed antiseptic product)**Xttrium** (Other Financial or Material Support, Conducted clinical trials and studies in which participating hospitals and nursing homes received contributed antiseptic product) **Syma Rashid, MD**, **Medline** (Other Financial or Material Support, Conducted studies in which participating hospitals received contributed antiseptic product)**Stryker (Sage**) (Other Financial or Material Support, Conducted clinical trials and studies in which participating hospitals and nursing homes received contributed antiseptic product)**Xttrium** (Other Financial or Material Support, Conducted clinical trials and studies in which participating hospitals and nursing homes received contributed antiseptic product) **Taliser R. Avery, MS**, **Medline** (Other Financial or Material Support, Conducted studies in which participating hospitals received contributed antiseptic product)**Molnlycke** (Other Financial or Material Support, Conducted studies in which participating hospitals received contributed antiseptic product) **Kenneth Sands, MD, MPH**, **Medline** (Other Financial or Material Support, Conducted studies in which participating hospitals received contributed antiseptic product) **Julia Moody, MS**, **Medline** (Other Financial or Material Support, Conducted studies in which participating hospitals received contributed antiseptic product)**Molnlycke** (Other Financial or Material Support, Conducted studies in which participating hospitals received contributed antiseptic product) **Kimberly N. Smith, MBA**, **Medline** (Other Financial or Material Support, Conducted studies in which participating hospitals received contributed antiseptic product) **Brandon Carver, BA**, **Medline** (Other Financial or Material Support, Conducted studies in which participating hospitals received contributed antiseptic product) **Caren Spencer-Smith, MS**, **Medline** (Other Financial or Material Support, Conducted studies in which participating hospitals received contributed antiseptic product)**Molnlycke** (Other Financial or Material Support, Conducted studies in which participating hospitals received contributed antiseptic product) **Russell Poland, PhD**, **Medline** (Other Financial or Material Support, Conducted studies in which participating hospitals received contributed antiseptic product) **Jason Hickok, MBA**, **Medline** (Other Financial or Material Support, Conducted studies in which participating hospitals received contributed antiseptic product)**Molnlycke** (Other Financial or Material Support, Conducted studies in which participating hospitals received contributed antiseptic product) **Arjun Srinivasan, MD**, Nothing to disclose **John A. Jernigan, MD, MS**, Nothing to disclose **Jonathan B. Perlin, MD, PhD**, **Medline** (Other Financial or Material Support, Conducted studies in which participating hospitals received contributed antiseptic product)**Molnlycke** (Other Financial or Material Support, Conducted studies in which participating hospitals received contributed antiseptic product) **Richard Platt, MD, MSc**, **Medline** (Research Grant or Support, Other Financial or Material Support, Conducted studies in which participating hospitals received contributed antiseptic product)**Molnlycke** (Other Financial or Material Support, Conducted studies in which participating hospitals received contributed antiseptic product) **Susan S. Huang, MD, MPH**, **Medline** (Other Financial or Material Support, Conducted studies in which participating hospitals and nursing homes received contributed antiseptic and cleaning products)**Molnlycke** (Other Financial or Material Support, Conducted studies in which participating hospitals and nursing homes received contributed antiseptic and cleaning products)**Stryker (Sage**) (Other Financial or Material Support, Conducted studies in which participating hospitals and nursing homes received contributed antiseptic and cleaning products)**Xttrium** (Other Financial or Material Support, Conducted studies in which participating hospitals and nursing homes received contributed antiseptic and cleaning products)

